# Draft Genome Sequences of Three *Mycobacterium bovis* Strains Identified in Cattle and Wildlife in France

**DOI:** 10.1128/genomeA.00410-17

**Published:** 2017-07-06

**Authors:** Maxime Branger, Amandine Hauer, Lorraine Michelet, Claudine Karoui, Thierry Cochard, Krystel De Cruz, Sylvie Henault, María Laura Boschiroli, Franck Biet

**Affiliations:** a INRA, Université de Tours, UMR 1282, Infectiologie et Santé Publique, Nouzilly, France; b Université Paris-Est, Laboratoire National de Référence Tuberculose, Unité Zoonoses Bactériennes, Laboratoire de Santé Animale, ANSES, Maisons-Alfort, France

## Abstract

*Mycobacterium bovis* is the etiologic agent of bovine tuberculosis, a chronic infectious disease affecting livestock, wild animals, and sometimes humans. We report here three draft genome sequences of *Mycobacterium bovis* strains of spoligotypes SB0821 and SB0134, isolated from wildlife but circulating in wildlife-livestock multihost systems, and SB0121, circulating exclusively in cattle.

## GENOME ANNOUNCEMENT

Bovine tuberculosis (BT) due to *Mycobacterium bovis* is a chronic infectious disease affecting domestic animal species, like cattle, wild animals, and sometimes humans (1–4). 

In France, an officially BT-free member of the European Union (EU), *Mycobacterium bovis* remains endemic in a few regions of the country (5). The in-depth genetic study of strains of *M. bovis* responsible for the persistence of outbreaks revealed several strains with a genotypic profile associated with localization to a specific region (6). Some of these strains infect both livestock and wildlife in an undifferentiated way and proved their ability to proliferate in multihost systems. To understand how they have evolved, strains of spoligotypes SB0821, SB0134, and SB0121 specific to the Aquitaine and Normandie regions were selected to perform whole-genome sequencing. Strain D11-01601 of spoligotype SB0821-MLVA 6 5 5 3 11 2 5.5 8, strain D11-03130 of spoligotype SB0121-MLVA 5 2 5 3 8 2 5 6, and strain D11-00843 of spoligotype SB0134-MLVA 7 4 5 3 10 4 5 10 were, respectively, isolated in Aquitaine (2011) from retropharyngeal lymph nodes of a wild boar and in Haute-Normandy (2011) and in Aquitaine (2011) from respiratory lymph nodes of cattle, as described before (6).

Isolates were grown in 10 ml of Middlebrook 7H9 liquid medium supplemented with 10% oleic acid-albumin-dextrose-catalase (OADC) for 4 weeks. Bacteria were thermolysed for 1 h at 80°C, and chromosomal DNA was extracted with phenol-chloroform.

DNA sequencing was performed at Genoscreen (Lille, France) on a HiSeq 2500 (Illumina, San Diego, CA, USA) to obtain 2 × 100 paired-end sequences, for an average coverage of 80×. Sequencing strain D11-01601 produced 3,238,397 paired-end reads of 100 bp and 3,085,262 reads after filtering with Sickle version 1.33. We performed *de novo* assembly using SPAdes version 3.9.0 (2) on trimmed sequences, with a k-mer size of 55. We obtained 146 contigs (largest, 237,066 bp) for a total length of 4,296,059 bp; we used only contigs larger than 200 bp, a G+C content of 65.54%, and an *N*_50_ of 98,742 bp (QUAST version 4.2 [7]). Annotation was performed by the National Center for Biotechnology Information (NCBI) Prokaryotic Genome Annotation Pipeline (8), which predicted 4,112 coding DNA sequences (CDSs) for 4,297 total genes, 45 tRNAs, and 134 pseudogenes.

As the same pipeline, strain D11-00843 sequencing produced 1,760,482 paired-end reads and 1,679,977 reads after filtering. We obtained 191 contigs (largest, 176,982 bp) for a total length of 4,294,610 bp (G+C content, 65.54%; *N*_50_, 59,829 bp). The annotation predicted 4,128 CDSs for 4,328 total genes, 45 tRNAs, and 149 pseudogenes. For strain D11-03130, sequencing produced 3,187,526 paired-end reads and 2,993,919 reads after filtering. We obtained 155 contigs (largest, 236,095 bp) for a total length of 4,308,841 bp (G+C content, 65.56%; *N*_50_, 122,015 bp). The annotation predicted 4,127 CDSs for 4,314 total genes, 45 tRNAs, and 136 pseudogenes.

### Accession number(s).

The whole-genome shotgun projects have been deposited at DDBJ/ENA/GenBank under accession numbers MTZQ00000000, MTZR00000000, and MTZS00000000 for strains D11-01601, D11-00843, and D11-03130, respectively. The versions described in this paper are versions MTZQ01000000, MTZR01000000, and MTZS01000000, respectively.

## References

[1] Amanfu W 2006 The situation of tuberculosis and tuberculosis control in animals of economic interest. Tuberculosis (Edinb) 86:330–335. doi:10.1016/j.tube.2006.01.007. 16644282

[2] Bankevich A, Nurk S, Antipov D, Gurevich AA, Dvorkin M, Kulikov AS, Lesin VM, Nikolenko SI, Pham S, Prjibelski AD, Pyshkin AV, Sirotkin AV, Vyahhi N, Tesler G, Alekseyev MA, Pevzner PA 2012 SPAdes: a new genome assembly algorithm and its applications to single-cell sequencing. J Comput Biol 19:455–477. doi:10.1089/cmb.2012.0021. 22506599 PMC3342519

[3] Cosivi O, Grange JM, Daborn CJ, Raviglione MC, Fujikura T, Cousins D, Robinson RA, Huchzermeyer HF, de Kantor I, Meslin FX 1998 Zoonotic tuberculosis due to *Mycobacterium bovis* in developing countries. Emerg Infect Dis 4:59–70. doi:10.3201/eid0401.980108. 9452399 PMC2627667

[4] Frothingham R, Meeker-O’Connell WA 1998 Genetic diversity in the *Mycobacterium tuberculosis* complex based on variable numbers of tandem DNA repeats. Microbiology 144:1189–1196. doi:10.1099/00221287-144-5-1189. 9611793

[5] Bekara ME, Azizi L, Bénet JJ, Durand B 2016 Spatial-temporal variations of bovine tuberculosis incidence in France between 1965 and 2000. Transbound Emerg Dis 63:101–113. doi:10.1111/tbed.12224. 24735092 PMC5858770

[6] Hauer A, De Cruz K, Cochard T, Godreuil S, Karoui C, Henault S, Bulach T, Bañuls AL, Biet F, Boschiroli ML 2015 Genetic evolution of *Mycobacterium bovis* causing tuberculosis in livestock and wildlife in France since 1978. PLoS One 10:e0117103. doi:10.1371/journal.pone.0117103. 25658691 PMC4319773

[7] Gurevich A, Saveliev V, Vyahhi N, Tesler G 2013 QUAST: quality assessment tool for genome assemblies. Bioinformatics 29:1072–1075. doi:10.1093/bioinformatics/btt086. 23422339 PMC3624806

[8] Tatusova T, DiCuccio M, Badretdin A, Chetvernin V, Nawrocki EP, Zaslavsky L, Lomsadze A, Pruitt KD, Borodovsky M, Ostell J 2016 NCBI Prokaryotic Genome Annotation Pipeline. Nucleic Acids Res 44:6614–6624. doi:10.1093/nar/gkw569. 27342282 PMC5001611

